# Uncomfortably numb: suicide and the psychological undercurrent of COVID-19

**DOI:** 10.1017/ipm.2020.49

**Published:** 2020-05-21

**Authors:** H. Hughes, M. Macken, J. Butler, K. Synnott

**Affiliations:** Department of Trauma & Orthopaedic Surgery, Mater Misericordiae University Hospital, Dublin, Ireland.

Dear Sir,

On an eerily quiet, urban Dublin street lies an apartment block, it’s balconies chequered with identical Irish flags carrying a defiant yet simple, national slogan; ‘You’ll Never Beat the Irish’. It is the brilliance not only of the green–white–orange against the brutalist background that catches the eye but also the boldness, the sheer determination of the silent protest. It is a taunt to the insidious international public enemy that is COVID-19. It first barrelled through Ireland’s door in late February 2020. On March 12, 2020, our Taoiseach Leo Varadkar addressed the nation from Washington, DC, issuing guidance on a nationwide lockdown (Radio Teilifís Éireann, 2020). The virus had not yet gripped the health of the nation, but it was certainly within range of its virulent vice-grip. He urged us to socially distance in order to slow the spread.

During times of crisis, we as humans have a natural tendency to seek solace in collectivism and reassurance in joint protection from a threat or crisis (Baumeister & Leary, 1995). Furthermore, our psychobiological response to stress is essential to activate our survival instincts as a species; fight or flight, as it were. But what happens when the one you wish to fight is invisible to the naked eye and the wingspan of your flight is capped by the four walls in which you reside due to social isolation? For those enduring stressful times, anxiety, distress and negative emotions are heightened when we find ourselves separated from those important to us (Baumeister & Leary, 1995). The uncertainty and unpredictability associated with COVID-19 has created a parallel pandemic of fear, anxiety and distress (Ammerman *et al*., 2020, Yao *et al*. 2020). Research from China has demonstrated heightened rates of psychological distress attributable to COVID-19 (Yao *et al*. 2020). However, the correlation between epidemics, mental health and suicide is not a new revelation. During the 2003 SARS-2 outbreak, China observed a sharp increase in the rate of suicide among its elderly population (Chan *et al*. 2006). Furthermore, illness-imposed quarantining has been associated with detrimental mental-health ramifications for those who must endure it (Brooks *et al*. 2020).

While COVID-19 alone may not be the sole impetus for suicidality, the associated social disconnection, physical isolation and routine disruption may be a pernicious cocktail of risk factors (Ammerman *et al*. 2020). Suicide is a leading cause of death world-wide (World Health Organisation, 2019). In Ireland, it was estimated that in 2018, one person died per day by suicide (Digital Desk Staff, 2019). According to recent research from the United States, 45% of individuals with suicidal ideation explicitly linked their thoughts to COVID-19 (Ammerman *et al*. 2020). The same study found that intentional exposure to COVID-19 is being used as a method of suicide (Ammerman *et al*. 2020). Preliminary data from Ireland’s National Spinal Injuries Unit demonstrate that, over a 2-month period from March to April, the number of admissions attributable to suicide attempts increased from 1.3% in 2019 to 17% in 2020. The psychological impact of COVID-19 serves as a wicked side-kick to its physical manifestations. There is a distinct need to bolster mental-health services around vulnerable populations. Both national and international bodies have published widely on this topic, offering guidance and support for those suffering in the side-lines (Aware, 2020, Mental Heatlh America, 2020).

In the words of Thomas Fuller, ‘if it were not for hope, the heart would break’ (Grayling, 2007). Hope is, perhaps, one of the core motivators in our gallant international efforts to overcome the crisis that is COVID-19. A subconscious drive to cling to life, a conscious strive to protect those we love propels our collective efforts in this time of separation. The hope that things will return to some semblance of normality, or, rather a ‘new-normal’, whatever that may come to look like is tangible. It hangs in the air. From the perspective of those who are at the brink of despair and desperation, hope is what may keep them alive; the hope that things may eventually change for the better. In the words of G. K. Chesterton, ‘hope means hoping when everything seems hopeless’ (Chesterton, 1990). The proverbial light in this time of darkness is shining for us all; only, some need help to see it. Our Taoiseach Leo Varadkar bravely and boldly stated, ‘we will prevail’ (Radio Teilifís Éireann, 2020). Indeed, we must.
